# The Treatment of Proximate Surfaces

**Published:** 1890-12

**Authors:** George A. Wilson


### THE TREATMENT OF PROXIMATE SURFACES.[1]

BY GEORGE A. WILSON, D.D.S.

To many of you it may seem presumptuous in me to attempt to offer a single new thought on the subject I have chosen for my paper this evening.

While I cannot hope and do not undertake to add much to what is known, I still think it is not too late to make it interesting.

Repetitions may therefore be pardonable if we review some of the laws that govern these operations and endeavor to see if they are understood and complied with.

I suppose it is because I so love the work and have lived in it so long that the question has become one of first importance. It is because I believe that to every conscientious worker it is a theme of ever-recurring interest; it is one which never fails to appeal to our best judgment and challenges our finest skill; it is one whereon the most proficient among us welcomes more light and knowledge in methods of treatment and the establishment of correct principles of practice.

To this end—viz., the firmer establishment of principles and ideal methods, and thoroughly practical as well—I would engage

---

[1] Read before the New York Odontological Society, October 21, 1890.

your thoughts this evening, for I believe the time has come when we should no longer be primarily seekers after the best ways of practice, but proficient workers on principles which are concurrent and coexisting with accepted truth and law.

The correct treatment of proximate surfaces in teeth brings us face to face with a very complex problem, but, like all much-mixed equations, we must first reduce it to a simple one.

In considering this matter I must perforce do so from a standpoint which twenty years' experience has proved to me to be the correct one.

Beginning as a disciple of contour work in the office of my honored preceptor, Dr. Atkinson, and having the advantages of the experience of such brilliant operators as Varney, Palmer, and Webb to aid me in my first researches after the best way, it is no wonder that I then and there espoused the newer system to which I have ever since given my unwavering allegiance. In fact, I may well consider myself fortunate in coming on to the stage of action just at that time and under so favorable circumstances, and perhaps do not deserve as much credit for steadfastness as many others whose chances, as students, were hedged in by meagre and, in many cases, adverse opportunities.

Aside from the fact that my whole professional life has been singly devoted to the development of this newer system, I have no plea to offer in support or defence of contour work as the correct principle. At this time it needs none. We are workers on accepted and well-grounded principles, having passed that time when theory and argument preceded and often defeated intelligent effort. The voice and work of the majority of the best men in the profession make a plea more convincing and eloquent than a Varney or a Webb could offer were they among us.

Were reasons asked by young men entering the profession why they should adopt this better way, first among them would be, it is in support of nature's plan: this plan, as manifest throughout the animal world, so far as the shaping and building of teeth is concerned,—leaving out that subsequent factor, decay,—is not to be questioned or improved upon. Their arrangement and adaptation for the work designed for them to do is as perfect as divine wisdom could make it.

Another reason is the inconsistency and futility of arraying our skill against the ravages of decay while we array ourselves against nature as well.

Decay and the causes of decay, perhaps arising from serious infringement of the laws underlying the preservation and integrity of the teeth, come in, and here our work begins. The charm and fascination of feeling that everything one can do to prevent, arrest, and redeem the teeth from the ravages of decay and aid nature in restoring and sustaining her ideals may ever be counted upon as a most potent aid and inspiration. To feel one's self able to give back to suffering fellow-mortals what decay has robbed them of—comfort, consciousness of a wholeness and restoration of first forms and first unconsciousness of loss and pain—is certainly a high moral incentive for every dentist, young or old, to consider.

Hitherto, it seems to me that the contour system of filling teeth has been discussed far too much from the stand-point of restoration, while the equally important principle of prevention and the establishment of conditions which render subsequent attacks of decay far less liable to occur has been overlooked.

Claims for superiority in this particular over the old system have not been urged and maintained by our best writers on this subject as persistently or as justly as they have a right to do. Very many of the principles involved in a perfected system of dentistry, as applied to the natural teeth, have been but lightly touched upon. Obliteration of interproximate spaces, deranged occlusion, perfect lines of axis of the teeth, preservation of the perfect arch and consequent freedom from pressure on tissue around the teeth,—these are all matters which have received far less attention than the contour filling itself. Against this the bolts of the opposition have mainly been shot. The old system was open to all these dangers and invited them; the new anticipated and prevented them.

The fatality of making spaces between the teeth, as a rule of practice, is seen and known, and the practice, as Dr. Black tells us, has passed into practical oblivion among almost all of the best men in the profession. It is, and always was, an indulgence in easy and crude methods fraught, as every honest man knows, with a train of evils which he does not care to have brought to his door. Earnestness—even enthusiasm—in this matter need not constitute a plea for superiority, but rather looks to execution more perfect and methods reduced to harmony with natural and mechanical laws, better suited to cope with ever-varying conditions which make our work necessary.

Just here let me say that I am pleading for the adoption of better rules of practice, not speaking of individual and particular cases. I would not be understood as saying that strict contour work was in every case practical or feasible. Limitations and environments of many kinds render this impossible in certain cases. I refer to that class of cases which invites the exercise of our best skill and puts no restrictions thereon. It is in this domain that I would seek to establish the highest standards.

Referring to recent literature on this subject, and notably the papers of Professor Black, Drs. Jack and Perry, we have the subject handled in a most masterly way. Dr. Perry, especially in his treating of the matter published in the INTERNATIONAL DENTAL JOURNAL of last year, evidently meant that there should not be anything left to be said. Certainly he has given us with charming candor—peculiar alone to him—the "logic" of his varied experience. No more cogent or conclusive argument need be asked for—were such a defence necessary—in support of the contour principle. First an enthusiastic contourist, then a lapse into the middle ages, a "dark period" of doubt and distress, but finally to emerge again into the light, one of the ablest exponents, both by word and deed, of the better way.

At the last anniversary of the First District Society of this city a paper was read by Dr. Allan, of this Society, and discussed by the representative men there present. As many of you may remember, that discussion was a very one-sided one and constituted as eloquent a plea as possible for the superiority of the new over the old system, theories, and methods. Dr. Allan, in starting, said, "The bulk of the practice of the day is in the directions of face-fillings; the theory of the day is contour fillings." Had this sentiment been uttered twenty-five years ago it would have better fitted the times. The sentiment expressed in that discussion showed most conclusively that the profession had outgrown those crude and gruesome methods. As Dr. Allan promises to be heard from again (and certainly needs to be, to set himself right) on this subject, I refrain from further criticism.

Referring to some mechanical principles which have aided me greatly in reproducing contour, I must go back and criticise Dr. Perry a little. I can find no mention in his paper of a principle that gold could be so inserted in a tooth as to strengthen the walls rather than weaken them.

Hitherto, those who have opposed this method have agreed that contour work weakened the tooth. I allow that in certain cases it may, but as a rule it need not, but, on the contrary, it can be so done as to strengthen and hold together very frail walls, relieving them of all strain, while the gold takes the wear and work. We may call it the bevel-edge principle.

Then Dr. Perry conveys the idea that enamel has great strength as seen in the "enamel rim," "the great arch," "the coronal arch," etc.; he would spare it, trust it to do what nature, in my judgment, never intended it to do. His argument here is, I think, misleading.

There is great strength and resisting power in enamel only when force is applied to it endwise. Nature so arranged those rods on teeth that from any direction assaults of an enemy were met endwise; reverse this law and flank it, attack it from its side, and it splits as easily as match-wood. Hence I say, as a cardinal principle, never trust enamel to hold gold or other metal; cut till you get anchorage into material which is built on a different plan and has holding power and strength; dentine, Dr. Perry's "great arch," as I understand it, forms the bridge over cavities in proximate surfaces where decay has not proceeded far enough to warrant cutting through from the grinding surfaces.

This class of fillings, where this arch was left, have, with me, been more productive of failures than larger ones where it was cut away. I have found that this bridge of enamel would give way sooner or later, and I would have to do what I should have done at first,—cut through from the grinding surface and into the fissure as well, perhaps.

Dr. Perry also tells us that "enamel does not coalesce in the fissures." How, then, can it be of much service in binding the cusps together? The dovetail or opposing-point principle in shaping cavities to hold gold rather than retaining pits,—anchorage into dentine,—and, when practical or necessary for future strength, bringing the gold over into the fissures and grinding surface, forming a knee-shaped filling which relieves the proximate walls of all strain, and bevelling these edges to prevent their escape,—as the jeweller his diamond,—are some of the principles upon which contour work can be successfully done.

There is another important thing to consider in beginning and ending an operation of the proximo-contour character, and that is, first, to note the conditions and positions of those parts of teeth affected by caries; and, last, that when the work is completed a new set of conditions is established, new relations of the parts subject to decay, and free margins which can be examined easily and quickly.

If enamel must be left at the cervical border, trim it smoothly, slightly round or bevel the edge, and place and pack the first pieces of soft gold with hand pressure. No malleting is allowable here until a thickness of gold sufficient to protect the thin rim of enamel from fracture by the mallet is secured.

Dr. Perry speaks of the "matrix" as an adjunct to perfect contour filling, but, as I am pleased to see, with evident misgiving.

Does the artist need a stencil-plate for his beautiful fresco and decoration? the sculptor a mould into which to press his clay for a model of the form which is to follow? No. The true artist who rebuilds and restores the forms of teeth needs only the trained eye and skilled hand.

There is one prime necessity,—a dentist should be born such. As Oliver Wendell Holmes would tell us, he must have the cumulative qualities of four generations of mechanics concentrated within himself, and out of this wealth of endowment he can formulate a code of dental mechanics without great study or need of imitation. The true artist does not depend on the work of others for his models or inspirations. He may use appliances and methods in pursuing his work which others have found to be good, but he accepts their use because they fall in line and come within the scope of his own work.

In conclusion, let me say just a word in honor of the men who, for half a century or more, have lived in the light and helped others to grow towards the sunshine, and who honor us with their presence to-night,—Drs. Atkinson and Dwinelle.

As Dr. Perry has so eloquently put it, let us all congratulate them that they have lived not only to see the "irresistible tide of ripened professional judgment" setting in their direction, but rising and swelling about them and bearing them on with ever-increasing honors to and beyond the end of their earthly career.

Let us be proud of and enjoy them while we may, as our beloved masters and teachers, workers ever and always in establishing the highest standards, and never content until attained. Well may they be proud for their part in building up a system of dentistry, which, "built upon the needs of human kind, will endure while those needs last."

